# Genito - Urinary and Syphilitic Diseases

**Published:** 1902-03

**Authors:** Byron H. Daggett

**Affiliations:** Buffalo, N. Y.


### PROGRESS IN MEDICAL SCIENCE.

### Genito - Urinary and Syphilitic Diseases.

CONDUCTED BY BYRON H. DAGGETT, M. D., Buffalo, N. Y.

### CLINICAL NOTES ON GLEET.

RAROGLI (*New York Medical Journal*, January 4 and 18, 1902,) says that the name gleet is a popular term, but the different treatises on genito-urinary disease name this affection chronic urethritis.

This disease is a complex of different conditions. It is a chronic inflammatory condition, located in some part of the canal or its adnexa. From the gonorrheal infection to the formation of stricture there is a continuation of disorders and of alterations of tissues, due to the derangement of nutrition resulting from the infection. If a man has a stricture twenty years after having acquired a gonorrhea, it would be called an evolution of the gonorrheal process. General gonorrheal infection may occur, but this condition is very rare. It remains limited in most cases to the mucous membrane of the genitals or to the organs in direct relation.

The gonococci may remain dormant for a long time, in Littre's glands, in Morgagni's crypts, Cowper's glands, the prostatic ducts, and the seminal vesicles, without disclosing their presence. They may be aroused from this condition of latency by hyperemia of the parts caused by alcohol or venery. In this case there is a fresh outbreak, simulating a new invasion. This is autoinfection. In most cases, after a few years, the germ is dead so that infection does not occur.

*Symptomatology.*—A scanty, whitish fluid appears in the morning; shreds are found in the urine, or it may be turbid.

*Subjective Symptoms.*—Occasionally a patient complains of a burning sensation in the act of micturition, or of pain from ejaculation. Some complain of transient pains in the lumbar and genital regions, others of fatigue. Others suffer from a morbid nervous superexcitability extending over different nerve territories, constituting sexual neurasthenia. Chronic posterior urethritis causes nervousness, loss of flesh, nocturnal emissions, sleeplessness, mental depression, incomplete erections, retarded ejaculation and impotence or premature ejaculation. Often the cause of neurasthenia is revealed by the passage of a sound.

*Physical Examination.*—The morning drop must be microscopically examined, although the result does not explain the reason of the stubbornness of the affection. We find permanent epithelium, mucin and uninuclear leucocytes. At times a few cocci are found, at other times they are abundant. The gonococci are seldom found in the urine or in the discharge. Finger says that the stubbornness of the discharge is owing to the affection of the prostate. The prostate should be massaged and the secretion examined for gonococci, which, if present, indicate infectiveness.

Milking of the prostate does not generally bring forth secretion and examination of the secretion does not often reveal anything abnormal, yet the urethral drop is always suspicious.

There is no doubt that gleet has its seat in the posterior urethra and continues for years in a state of chronic hyperplastic inflammation, producing sclerosis and shrinking of the tissues, forming stricture.

Littre's glands lie in the membranous portion of the urethra; they are hiding places for the gonococci and foci of infection. Epithelial débris blocks the excretory ducts of the urticles and glands and prevent the access of the remedy to the interior. These plugs, escaping from the excretory ducts float in the urine and form the comma-like filaments of Fürbinger. In every case of gleet the prostate should be examined through the urethra and rectum. When the sound is removed a little drop of milky fluid follows, which shows small leucocytes, large epithelial cells, amorphous uric salts of decomposed urine, which cause maceration, infiltration and granulation. This condition will produce stricture after a time. Chronic gonorrheal inflammation produces hypertrophy of the connective tissue and changes in the epithelium. In acute cases the cells degenerate; in chronic cases they proliferate and become hypertrophied. These changes modify the elasticity of the urethra, causing functional disturbance and prevent the squeezing out of any drop of urine. This residual urine continues the inflammatory process.

*Pathology.*—When the gonococcus lodges on the mucous membrane it develops, proliferates and causes a reaction with all the symptoms of inflammation. This constitutes acute gonorrhea. The urethral mucous membrane is a fertile soil for their development. They infect the surface by continuity and by contiguity, enter the glands in the lacunæ and the organs connected with the affected mucosa.

It appears that the gonococci remain near the surface. Brunner says that the gonococci quickly penetrate the epithelium and collect in great numbers in the lymph spaces of the upper connective tissue layer. Halstead says that although large numbers of gonococci penetrate the tissues, yet their development takes place near the surface of the mucous membrane.

Tuton, Jadasson, Fabry, Dinkler and Rosinsky claim that gonococci multiply on the surface of the pavement cells and only penetrate the superficial layers of the mucosa. They are found in the cylindrical epithelium, near glands and in the excretory ducts and glands. They only accidentally affect connective tissue. Several investigators have found these germs in abscesses and in edema of the prepuce. Gonococci persistently abide upon mucous membrane in a latent state and produce chronic manifestations. They lose virulence by repeated reproductions on the same ground and only produce chronic manifestations, but irritation or emigration to a new field will arouse their pristine vigor.

The urethroscope shows the mucous membrane dark red, covered with small red points which easily bleed. The infiltrated mucosa protrudes into the urethroscope. White patches may be seen, due to thickening of the epithelial layer. Sometimes nodules, the size of a pin head, are found. They are due to occlusion and cysts of the lacunæ glands. Macroscopical examination does not show tissue change of chronic inflammation. Microscopical examination shows small cell infiltration and connective tissue proliferation in the subepithelial structure (Finger). Such infiltration is usually limited to patches of different sizes in the neighborhood of the lacunæ and glands, and this condition causes the granular appearance found with the urethroscope. Chronic urethritis preferably attacks the bulb and the junction of the membranous and prostatic divisions of the canal. The evolution of this process produces fibrous or cicatricial tissue which, becoming sclerotic, forms organic stricture. Infiltration is the first stage of the formation of stricture. Examination of the prostate, seminal vesicles and Cowper's glands should always be made for diagnosis and treatment of gleet.

*Prognosis.*—An old gleet, complicated with chronic prostatitis can be much improved by well-directed treatment, but complete recovery in some cases is very difficult to obtain. Much patience is required on both sides. The patient must observe strict regimen, must avoid liquor and venery. The prognosis is more favorable in recent cases and in cases uncomplicated with stricture and nervous symptoms.

*Treatment.*—Treatment requires good judgment in the selection of the means to be employed. Internal treatment must be associated with local application. Urinary antiseptics, such as salol, salicylates, eucalyptus, sandal oil, boracic acid, the benzoates and urotropin. Bromides and opiates for relieving pain and neurasthenic conditions. Tonics, such as the hypophosphites, iron and strychnia. Local treatment is the most reliable. Irrigate the urethra, with or without the catheter. The recurrent catheter is the most useful in chronic posterior urethritis. Lavage alone seldom cures this condition. Instillation of silver and other remedies are often useful.

*The Urethroscope.*—The affected surface is brought into view by means of the endoscope and cotton is wrapped about the end of the rod, saturated with a 3, 5 or 6 per cent. solution of nitrate of silver and applied to the diseased membrane, and the surplus is taken up by a dry tampon. (The rods and endoscope made by the Electro Surgical Instrument Co., of Rochester, are well adapted for this work.—Abstractor.) 3 to 5 per cent. solution of copper sulphate or trichloracetic acid are no better than the silver. If the lesions are superficial, like erosions or granulations, a few applications will cure them. Begin treatment with lavage and apply through the endoscope a few applications of a 5 per cent. solution of silver protein or nitrate to the granulations will heal them in a short time. If deep infiltrations are present, although the erosions heal, the infiltrations remain and will run a progressive course and end in stricture.

*Mechanical Treatment.* — Reabsorption of the infiltrated patches is promoted by pressure with sounds and dilatation; with the urethrometer measure the calibre of the urethra and find the points of a forming stenosis and narrowing of the urethral diameter. Sounds should be introduced once a week and left in for five minutes. Medicinal remedies are also applied in various ways to the stenotic points to hasten reabsorption. Application of unguents have not given expected results, although this treatment seems rational. Application of electrolysis with a current of from 12 to 20 milliampères is a very satisfactory procedure. Otis, Oberlander and Kollman have improved these instruments until they are about perfect.

The dilatation must be carefully done under a 2 per cent.

solution of cocaine or eucaine. The endoscope shows as a result of dilatation small, longitudinal fissures in the infiltrated surface, which promptly and completely heal without a scar. This procedure begets rapid reabsorption of the infiltration. Dilate once in ten days to two weeks. Complete the treatment with the steel sound.

---

MERCURY—ITS ACTION UPON THE SYSTEM.

PORTER, W. H. (*Reprint from the Post Graduate*) claims that mercurials act upon the hepatic function in three successive, distinctive stages: (1) as a stimulant, (2) greatly increasing the flow of bile, (3) greatly decreasing the flow of bile.

There is no satisfactory chemical or physiological explanation of the action of this drug, yet its beneficial effects cannot be disputed. It is an inorganic substance, hence is not decomposed within the animal organism and does not form other compounds therein. Calomel is practically insoluble and comparatively nonirritating, yet it is a most active cathartic. Being nonirritating to the intestinal mucous membrane, it passes along the alimentary tract, and is absorbed into the entero-hepatic circulation and is carried up to the liver, where it acts as an irritant to the hepatic cells, which by their secretive action and purpose protect the system against foreign invasion, pick up the little particles of calomel from the blood and pass them along into the capillary bile ducts.

If the amount of calomel is small, it stimulates the cells to a little greater activity and the secretion is converted into the salts of the bile acids. This is physiological action. Larger quantities of calomel augment the amount of the bile acids and pigment, more than can be neutralised by the alkaline phosphates and carbonates and the excess causes cholagogue action. This action is temporary, as it soon exhausts the protoplasmic chemical activity of the cells. Thus, mercurials are stimulating, cholagogue and depressant.

The hypersecretion of the bile acids and its effect upon the intestinal tract is a pathological process. By means of this abnormal functional activity of the gland, nature rids the system of offending substances. In this way microbic ptomaine or toxic poison is eliminated or destroyed.

In like manner it aids as an antisyphilitic remedy without assuming that it is a direct chemical antidote to the syphilitic poison. We have no definite knowledge of the exact character of this poison. The mercurials augment the glandular and nutritive activity of the system and raise the physiological tone to such a degree that the system rids itself of toxic material.

Mercurials stimulate the hepatic cells; produce cholagogue action; are sedative by reason of over stimulation and secretion. They are alterative, antiphlogistic, antisyphilitic and diuretic. Ptyalism is due to the inactivity of the hepatic cells, the salivary glands attempting to do what should have been done by the hepatic cells.

